# Cemented dual-mobility total hip arthroplasty cups in a custom-made acetabulum: a clinical and radiological evaluation

**DOI:** 10.1051/sicotj/2025049

**Published:** 2025-08-26

**Authors:** Nicolas Blum, Guillaume Mesnard, Cécile Batailler, Sébastien Lustig

**Affiliations:** 1 Orthopaedic Department, Lyon North University Hospital, Hôpital de La Croix Rousse, Hospices Civils de Lyon 103 Grande Rue de la Croix Rousse 69004 Lyon France; 2 Orthopaedics and Traumatology, Univ Besançon, UFR Santé 19 rue Ambroise Paré 25000 Besançon France; 3 Univ Lyon, Claude Bernard Lyon 1 University, IFST-TAR, LBMC UMR_T9406 69622 Lyon France; 4 LIBM – EA 7424, Interuniversity Laboratory of Biology of Mobility, Claude Bernard Lyon 1 University 69622 Lyon France

**Keywords:** Hip arthroplasty, Custom-made acetabulum, Dual mobility cup, Cemented

## Abstract

*Background*: Acetabular reconstruction during revision total hip arthroplasty (THA) with major bone loss is a complex surgical challenge. The combination of custom-made (CM) acetabular components with cemented dual mobility (DM) cups may improve postoperative outcomes in this context. This study aims to assess the clinical, functional, and radiological results of this surgical approach. *Methods*: We conducted a retrospective, single-center observational study including 16 patients (mean age 70 years) who underwent revision THA between May 2016 and December 2024 using a cemented DM cup in a CM acetabular component. All patients presented with Paprosky 3A or 3B defects, and 38% had a history of periprosthetic joint infection (PJI). Functional outcomes were measured using the Oxford Hip Score (OHS) and modified Harris Hip Score (mHHS) pre- and postoperatively. Radiographic assessment included measurement of the center of rotation (COR) deviation in both axes, as well as acetabular inclination and anteversion on postoperative CT scans. Implant survival was analyzed using Kaplan-Meier methodology. *Results*: At a mean follow-up of 16.2 months, overall implant survival was 75%, increasing to 93.8% when excluding isolated DM cup revisions. No postoperative infections were observed. OHS improved from 14.1 to 27.6 and mHHS from 27.4 to 52.7 (*p* < 0.001 for both). A significant negative correlation was observed between vertical (*y*-axis) COR deviation and functional scores (*p* < 0.01), highlighting the importance of restoring vertical COR. Mean inclination and anteversion were 41.2° and 29°, respectively, generally within target alignment zones. *Discussion*: The combination of cemented DM cups with CM acetabular components appears to be an effective technique in complex revision THA. Functional recovery and implant survivorship are consistent with the existing literature, and the absence of infection despite prior PJI history suggests benefit from a multidisciplinary approach. Restoration of vertical COR is a predictor of functional outcomes.

## Introduction

Total hip arthroplasty (THA) revisions are challenging surgeries that have a significant impact on patients’ health and function [1]: their number is increasing each year and will continue to grow due to the constant rise in the number of THA being performed [2]. The growing number of cases inevitably results in an increased incidence of complex situations, particularly following multiple revision procedures, which can lead to substantial bone loss [3]. Acetabular bone loss is a major preoccupation [4]. Indeed, a significant bone defect with pelvic discontinuity sometimes necessitates the use of a reinforcement device. This can be evaluated with the help of the Paprosky classification system [5]. Antiprotrusio cages are devices of choice used for the management of massive acetabular defects, but their use is associated with a high rate of failure [6]. Custom-made (CM) acetabular implants have been recently developed to face anatomical complexities that may compromise the use of conventional acetabular reinforcement devices [7, 8]. This situation is most encountered during prosthetic revisions or, less frequently, in a tumoral context. These surgeries, nevertheless, often present a high risk of prosthetic dislocation, which justifies the use of dual-mobility (DM) cups for whom a reduction of postoperative instability has already been proven [9]. As some evidence suggests that the cementation of DM cups is a reliable option for revision arthroplasties, as they show low rates of loosening or dislocation [10], it could be extrapolated to their use in custom-made acetabular implants.

The purpose of this study was to assess the clinical, functional, and radiological outcomes after the use of cemented dual-mobility cups in a custom-made acetabular implant for the treatment of bone defects.

## Material and methods

### Study design

This is a single-center, observational study that retrospectively reviewed all the patients who underwent THA with the use of a cemented DM cup in a CM acetabular component, from May 2016 to December 2024, with a minimum follow-up of 6 months.

The exclusion criteria were an age under 18, pregnancy, or death occurring within 6 months after surgery. We did not exclude patients with a history of THA infection, and the inclusion was made regardless of the indication of the surgery. All procedures were performed in accordance with the ethical standards of the institutional and national research committee, the 1964 Helsinki Declaration and its later amendments, or comparable ethical standards. Written informed consent was obtained from all patients.

### Preoperative data

Patient demographics are displayed in Table 1. The ASA score was determined by the anesthesia department of our center. Every patient underwent preoperatively a metal artefact reduction sequencing CT scanning of their whole pelvis to assess the bone defect. CT data was provided to the CM acetabulum manufacturer and studied by engineers to plan and produce the customized implant. Radiographs of the hip and pelvis were complemented by radiographs so that we could classify every hip thanks to the Paprosky classification system and plan the dual-mobility cup size (and the stem size, whether it was a bipolar revision) with our institutional software (TraumaCAD, Brainlab, Germany). Oxford Hip Score (OHS) and modified Hip Harris Score (mHHS) were calculated preoperatively and mentioned in the Electronic Medical Record (EMR).

**Table 1:** Patient characteristics.

No. of patients	16
Age (yr)	
Mean	70
Median	71.5
Range	44 – 87
Sex (no. of patients)	
Female	9 (56%)
Male	7 (44%)
Body Mass Index (kg/m^2^)	
Mean	26.1
Median	26.3
Range	19.1 – 31.1
ASA score (points)	
Mean	2.3
Median	2
Range	1 – 4
Side (no. of patients)	
Left	8 (50%)
Right	8 (50%)
Paprosky grade (no. of patients)	
3A	5 (31%)
3B	11 (69%)
Preoperative LLD (mm)	
Mean	−20.9
Median	−12.5
Range	−85 – +30
Preoperative OHS (pts)	
Mean	14.1
Median	12.5
Range	4 – 29
Preoperative mHHS (pts)	
Mean	27.4
Median	25.5
Range	5 – 50
History of infection (no. of patients)	6 (38%)
Previous surgeries (no.)	
Mean	3.2
Median	3
Range	1 – 6

### Surgery

The surgery was performed by senior orthopaedic surgeons, using a posterior approach. We noted the type and size of cup used, type of the acetabular component (brand), type and size of the stem, operating time, blood loss, and eventual perioperative complications.

### Clinical outcomes

The patients were clinically reviewed at 2 and 6 months during continued follow-up and all the clinical informations were strictly reported and available in the EMR. The outcomes measures were implanting survivorship, postoperative mechanical or septic complications, postoperative OHS and mHHS. Postoperative leg length discrepancy (LLD) and range of motion (ROM) were also reported. Implant survivorship was calculated with use of the Kaplan-Meier method, with all-cause re-revision as the end point. The OHS and mHHS were obtained through the regular follow-up. The OHS ranges from 0 to 48 points (with 48 representing the best possible outcome) and the mHHS ranges from 0 to 100 points (with 100 representing the best possible outcome).

### Radiological outcomes

**Figure 1:**
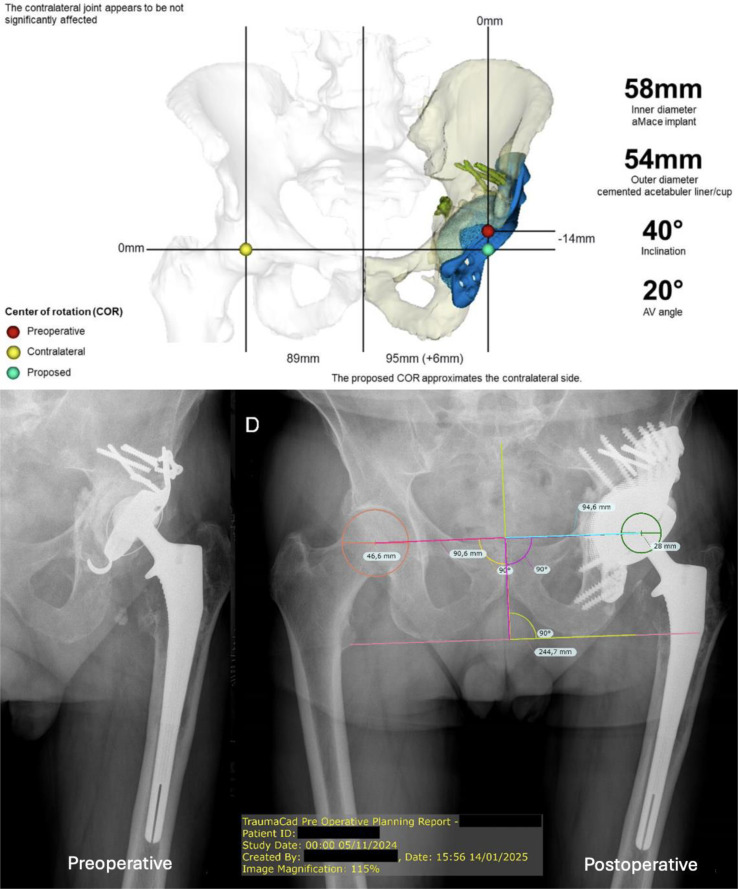
Complete case with 3D planification (Materialise) and radiographic measurement of *x*-axis and *y*-axis deviation of COR (case 10).

Every patient underwent preoperative radiographs with at least an anteroposterior (AP) view of the pelvis and a Lequesne’s profile. The same radiographs were done postoperatively. We then measured the mediolateral (*x*-axis) and craniocaudal (*y*-axis) deviation of the hip center of rotation (COR) in comparison with the contralateral hip (Figure 1). The COR was considered to be restored when the deviation was less than 5 mm in the relevant axis. Additionally, a CT scan with metal-reduction sequences was performed postoperatively to measure the acetabular anteversion and inclination, and to assess the host integrity or an eventual implant migration.

### Statistical analysis

Preoperative and postoperative OHS and mHHS scores were compared with the use of a paired Welch test. A Cox regression model and a Wilcoxon rank sum test were used to study the effect of selected prognostic factors on survival and functional outcomes. Statistical analysis was performed using SPSS 21.0 (IBM Corp., Armonk, NY, USA) with the level of significance at *p* < 0.05.

## Results

Seventeen patients were eligible, but 1 was excluded because they died within 6 months after surgery. We therefore included 16 patients (9 women and 7 men; sex-ratio: 0.78) with a mean age at surgery of 70 years (range: 43–87 years), a mean BMI and ASA score respectively of 26.1 kg/m^2^ (range: 19.1–31.1 kg/m^2^) and 2.3 (range: 1–4). The mean follow-up was 16.2 months. The LLD was measured at a mean of −20.9 mm (range: −85 to +30 mm) preoperatively versus −9.9 mm (range: −35 to 8 mm) postoperatively, which consisted of a mean reduction of 11 mm. Every patient had a history of at least one hip surgery, with a mean of 3, 2 surgeries (range: 1–6 surgeries), and 6 of them (37.5%) had a history of periprosthetic joint infection (PJI). We noticed 11 Paprosky 3B (69%) and 5 Paprosky 3A (31%) on the preoperative radiographs. Specifically concerning the surgery, eight patients (50%) underwent bipolar revision, 5 a unipolar revision (31.25%) and 3 (18.75%) a bipolar reimplantation. We mainly used Materialise custom-made implants (9; 56.25%), followed by Adler (6; 37.5%) and Mutars (1; 6.25%). The mean operating time was 214 min (range: 126–375 min). Average blood loss during the procedure was 877 mL (range: 300–2000 mL). Six patients (37.5%) underwent transfusion after the surgery. All the DM cups were Quattro (Lepine) implants, and their average diameter was 50 mm (range: 42–54 mm). We noticed one peroperative complication that consisted of a greater trochanter fracture, which was treated with cerclage.

### Clinical outcomes

**Figure 2:**
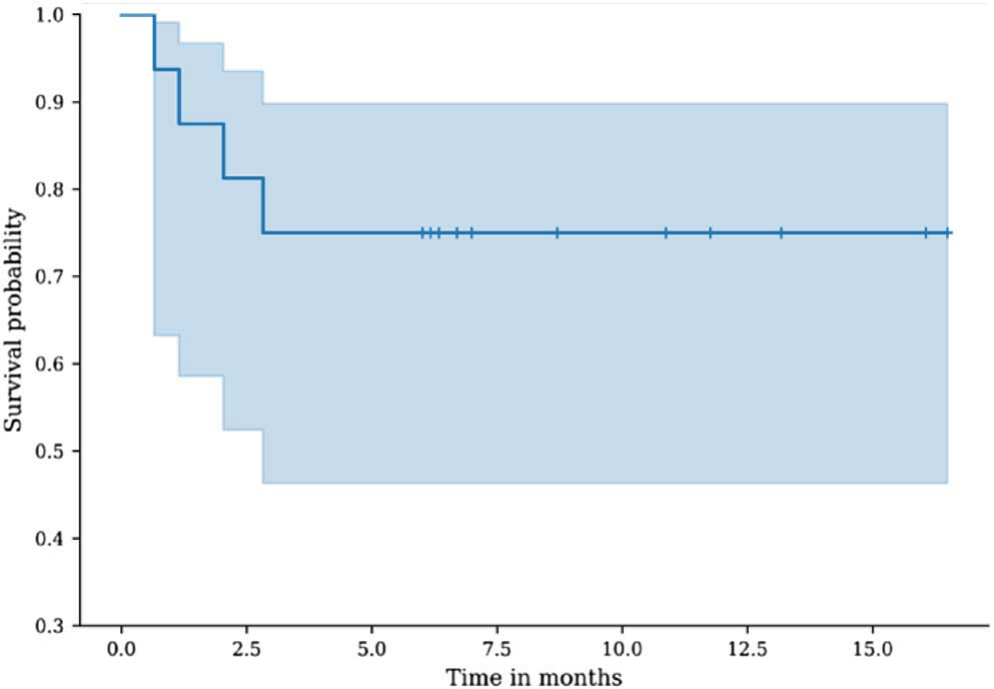
Kaplan-Meier curve demonstrating revision-free survival rates over the follow-up period.

Four patients suffered from mechanical complications leading to a failure: three dislocations and one aseptic loosening. All of these occurred during the first month following surgery and required reintervention (detailed in Appendix). The only aseptic loosening occurred following a placement failure and was revised with a Bursch-Schneider device 20 days after the index surgery. The three dislocations were revised through a cemented cup change and did not involve a recurrence of dislocation. These results equal a 75% survival rate (Figure 2). There was no septic complication despite the 38% history of periprosthetic joint infection. The OHS and mHHS improved significantly, respectively from 14.1 and 27.6 preoperatively to 27.4 and 52.7 postoperatively (*p* < 0.001), which consisted of a mean improvement of 13.5 points (+95.7%) and 25.3 points (+92.3%).

### Radiological outcomes

We calculated a mean deviation of 13.0 mm in the *x*-axis (range: 0.1–26.1) and 6.4 mm in the *y*-axis (range: 0.0–17.2). Only 2 patients (12.5%) were in a targeted deviation of 5 mm from the COR in the *x*-axis versus 8 patients (50%) in the *y*-axis. We did not measure the deviation of COR for the patient who sustained a CM implant replacement with a Bursch-Schneider device (case 2). The mean anteversion was measured at 29° (range: 10–39) and the mean inclination was measured at 41.2° (range: 36–52) on the CT scans. As well as the COR measures, we did not considerate the inclination and anteversion measures for case 2. Three patients (18.8%) were therefore unable to undergo postoperative CT scans, mainly for logistical reasons, as the scans were performed externally.

**Figure 3:**
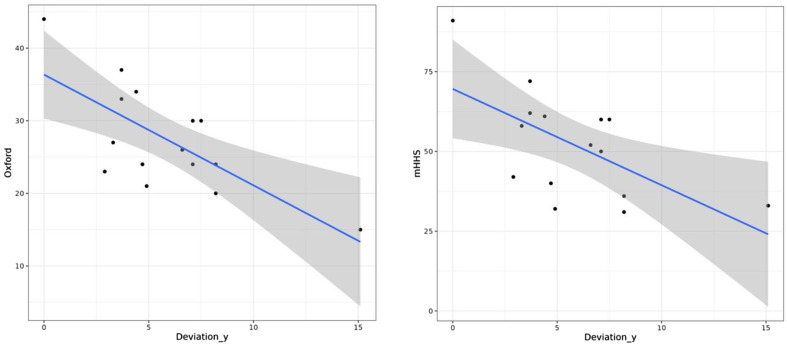
Graphs showing the negative correlation between y-axis deviation of the COR and postoperative OHS (p =0,031) (left) and the negative correlation between y-axis deviation of the COR and postoperative mHHS (p =0,018) (right).

**Figure 4:**
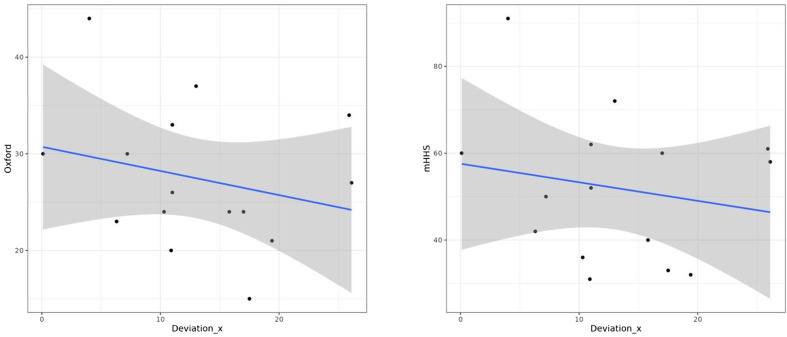
Graphs showing the negative correlation between x-axis deviation of the COR and postoperative OHS (p =0,49) (left) and the negative correlation between x-axis deviation of the COR and postoperative mHHS (p =0,67) (right).

We found a significative negative correlation between the *y*-axis deviation of the COR and the functional outcomes (Figure 3): the more the deviation of the COR on the *y*-axis was important, the less was the postoperative OHS and mHHS (*p* = 0.031 and *p* = 0.018 respectively). This was not significant on the *x*-axis (Figure 4). There was no statistical correlation between the measures of anteversion or inclination and the functional outcomes. We did not observe any statistical difference concerning survivorship in terms of radiological outcomes. All these results were described in Tables 2 and 3.

**Table 2:** Statistical correlation between radiological and functional outcomes (correlation coefficient is reported along with its associated *p*-value).

	OHS	mHHS
Anteversion	−0.429 (*p* = 0.16)	−0.372 (*p* = 0.23)
Inclination	0.0602 (*p* = 0.85)	0 (*p* = 1)
*X*-axis deviation	−0.192 (*p* = 0.49)	−0.120 (*p* = 0.67)
*Y*-axis deviation	−0.557 (*p* = 0.031)	−0.599 (*p* = 0.018)

**Table 3:** Statistical correlation between radiological outcomes and failure (mean is reported along with its associated standard deviation).

	Failure	No failure	*P*-value
Anteversion	36.5 (0.707)	27.5 (10.7)	0.24
Inclination	41.5 (7.78)	41.1 (4.82)	1
*X*-axis deviation	18.7 (7.52)	11.6 (6.97)	0.22
*Y*-axis deviation	5.83 (2.06)	5.83 (3.78)	0.66

## Discussion

This study demonstrates the effectiveness of the combination of cemented DM cups in a CM acetabular cup in terms of clinical and functional outcomes. Our survival rate of 75% at a mean follow-up of 16.2 months is similar to that in literature, as it varies from 60% to 100% [11]. However, we considered any revision as an end point, even if it only concerned the DM cup, which means this rate is underestimated: if we consider the revision of the CM component as the end point, this rate is 93.8%. De Martino et al. found an 82.7% rate of all-cause revision-free survivorship within 579 Custom Triflange Acetabular Components (CTAC) in 17 series, with an overall complication rate of 29% [12].

Our complication rate of 25% is in accordance with the literature in which it varies from 0.0% to 34.4% [11]. We observed three dislocations (18.8%): Tikhilov et al. reported a 13.1% rate whereas Myncke et al. calculated a 14% rate in 242 patients undergoing a CTAC [13, 14].

In terms of functional outcomes, we found a significant improvement in the OHS and mHHS scores. These findings were similar to other studies: Goriainov et al. [15] found a mean improvement of the OHS of 26.4 points (*p* < 0.0001), whereas Di Laura et al. [8] found 24 points (*p* = 0.0001). Concerning the HHS, this mean score improvement ranges from 23 to 40 points [16–18]. The improvement of functional outcomes seems to be influenced by the restoration of the COR on the *y*-axis, as we found a significant inverse correlation between the deviation of the COR on the *y*-axis and the functional scores. This result is interesting as there is no evidence in literature that a COR restoration failure is associated with bad functional outcomes. This could be because the few studies on the subject analyze only minimal deviations, whereas some patients in our study presented significant deviations that are more likely to have a clinical impact (up to 15.1 mm, as the limit is 5 mm) [19]. Only two of our patients had a COR in the targeted 5 mm on the *x*-axis, but there is no statistical evidence that a deviation of the COR on the *x*-axis is correlated with bad functional outcomes or survivorship. This great deviation on the *x*-axis is possibly due to the large thickness of the material, resulting in an offset increase that may, however, improve stability [20]. Indeed, positioning a DM cup inside a CM acetabular component creates a “cup-in-cup” effect, leading to an increase in the volume of implanted material.

The occurrence of an early loosening of the CM cup, which was due to a technical error, raises the issue of the technical difficulty with the slow learning curve, as it is a relatively rare and new surgery. This technical challenge can be explained by a difficult exposure due to multiple previous surgeries, which can cause disrupted anatomical landmarks and fibrotic tissues, whereas this type of large material requires an extended approach. The non-necessity to change the femoral stem in the majority of cases can also create an additional difficulty for exposure, and the use of new equipment involves a gradual familiarization. Goriainov et al reported a decrease in operating time and blood loss between the first 10 and subsequent 9 patients of their study, which reinforces this statement [15]. An extremely precise 3D-modeling allowing the production of a piece faithful to the anatomy should be associated to facilitate implantation and therefore reduce risks of mispositioning.

Our mean inclination and anteversion were respectively 41.2° and 29°. Lewinnek developed a safe zone that consisted of targeting values of acetabular anteversion and inclination in which the risks of postoperative instability were considered to be minimal (between 30° and 50° of inclination and 5° and 25° of anteversion) [21]. Reina et al. recently created a target zone thanks to CT-based criteria, between 40° and 50° of inclination and 15° and 30° of anteversion [22]. Considering that, we noticed that 58.3% of the measured inclinations and 16.7% of the measured anteversions were in that target zone (vs. 91.7% and 25% in Lewinnek’s safe zone). Freehand positioning of the acetabular component is challenging due to the loss of anatomical landmarks: in Choi et al.’s series of Paprosky 3B patients treated with conventional acetabular devices, the acetabular positioning Lewinnek’s safe zone occurred in only 56% of patients [23]. In Weber et al.’s study on Paprosky 3 defects treated with CM acetabular components, 3 of the 11 patients (27.3%) had an inclination above 50° or anteversion below 5° [24]. The mean inclination angle was similar to ours, with a mean of 48.6°, but we noticed a difference with their mean anteversion, which was 10.6°. This difference may be due to the fact that we measured the anteversion of the DM cup, rather than that of the CM component, which may be greater since we tend to manually add anteversion to an already anteverted component. This ability to adjust the anteversion and inclination of the DM cup after securing the CM acetabular cup represents an exciting feature, as it allows for the correction of any potential malalignment of the CM cup, the intentional addition of anteversion in patients at risk of dislocation, or even the easy repositioning of the DM component later if reoperation is needed. We did not find any statistical correlation between inclination and anteversion values and the occurrence of a dislocation. These results are biased because all the patients who had a dislocation underwent an acetabular revision, and the CT measures were made after that re-revision.

Finally, the absence of any septic complication despite the presence of a septic history in 38% of the cases is very encouraging and suggests that a PJI history should not be an obstacle. It also highlights the importance of a multidisciplinary approach, as every case with a PJI history has been preventively discussed with the infectiologists within a PJI multidisciplinary team (MDT). If a patient had a history of infection on the operated hip, this led either to a two-stage management approach, initially involving a debridement, antibiotics, and implant retention (DAIR) procedure, followed by a second reimplantation surgery at a later time, or to a one-stage approach with DAIR and CM acetabulum implantation. It has been demonstrated in the literature that a multidisciplinary approach reduces the risk of failure in the context of chronic THA infections, as suggested by Biddle et al who found a significative difference in failure rates between the group whose patients was discussed by a MDT and the group who did not (6.67% vs. 41.38%; *p* = 0.001) [25].

This study has several strengths and limitations. Firstly, the dual radiological follow-up using both X-rays and CT scans allowed for an accurate assessment of the radiological outcomes. Secondly, standardizing of the surgical technique for each patient helped reduce performance bias. Unlike many studies that exclude patients with a history of PJI, this cohort includes them, enhancing the generalizability of the findings and demonstrating the feasibility of this technique in high-risk patients. However, the cohort includes a limited number of patients, although it is challenging to build a large single-center cohort due to the high cost, the novelty, and complexity of the procedure, which restricts its practice to a few specialized centers. The minimal follow-up of 6 months with a mean of 16.2 months is not sufficient to conclude long-term outcomes. In patients who experienced early dislocation and underwent re-revision, postoperative CT-based alignment parameters were taken after the second surgery, potentially introducing bias into correlation analyses with outcomes.

## Conclusion

The use of cemented DM cups in CM acetabular implants appears to be an effective approach for restoring function in patients with severe acetabular bone defects. However, the restoration of the COR, particularly along the *y*-axis, emerged as a critical factor influencing functional outcomes. This highlights the importance of precise preoperative planning and surgical execution to optimize implant positioning. Further studies with larger cohorts and longer follow-ups are needed to validate these findings and refine surgical techniques.

## Data Availability

The data that support the findings of this study are available from the corresponding author upon reasonable request.

## Appendix

**Table A1:** Postoperative clinical outcomes.

Case	Follow-up (months)	Complication	Re-revision	Postop. LLD (mm)	OHS (points)	mHHS (points)
1	11.9	–	–	−15	24	36
2	101.9	Loosening	Unipolar revision with Bursch-Schneider	0	30	63
3	16.7	–	–	0	24	40
4	24	Dislocation	Unipolar revision of the cemented cup	−10	34	61
5	11	–	–	−20	26	52
6	13.4	–	–	−3	37	72
7	16.3	–	–	+5	30	50
8	6.7	–	–	−30	27	58
9	9.4	Dislocation	Unipolar revision of the cemented cup	−5	20	31
10	6	–	–	0	44	91
11	11.7	–	–	0	15	33
12	7	–	–	0	33	62
13	8.7	–	–	−35	24	60
14	6.2	–	–	−10	30	60
15	6.3	–	–	+20	23	42
16	6	Dislocation	Unipolar revision of the cemented cup	0	21	32

**Table A2:** Postoperative radiological outcomes.

Case	*X*-axis deviation (mm)	*Y*-axis deviation (mm)	Anteversion (°)	Inclination (°)	Osteointegration issue
1	10.3	8.2	–	–	–
2	–	–	–	–	Stem focal loosening
3	15.8	4.7	32	40	–
4	25.9	4.4	–	–	–
5	11	6.6	16	44	–
6	13	3.7	27	37	Stem focal loosening
7	7.2	7.1	13	43	–
8	26.1	3.3	36	40	–
9	10.9	17.2	37	36	–
10	4.0	0	10	40	–
11	17.5	15.1	30	35	–
12	11	3.7	35	43	–
13	17	7.1	–	–	–
14	0.1	7.5	39	37	–
15	6.3	2.9	37	52	–
16	19.4	4.9	36	47	–
